# Is ulnar shortening osteotomy or the wafer procedure better for ulnar impaction syndrome?: A systematic review and meta-analysis

**DOI:** 10.1097/MD.0000000000035141

**Published:** 2023-09-29

**Authors:** Joong Won Ha, Young Woo Kwon, Sujung Lee, Hyunsun Lim, Jinho Lee, Chae Kwang Lim, Jun-Ku Lee

**Affiliations:** a Department of Orthopaedic Surgery, National Health Insurance Service Ilsan Hospital, Goyang, South Korea; b Department of Orthopaedic Surgery, Yonsei University College of Medicine, Seoul, Republic of Korea; c Department of Orthopedic Surgery, Uijeongbu Eulji Medical Center, Uijeongbu-si, Gyeonggi-do, South Korea; d Medical Library, National Health Insurance Service Ilsan Hospital, Goyang, South Korea; e Department of Research and Analysis, National Health Insurance Service Ilsan Hospital, Goyang, South Korea.

**Keywords:** ulnar impaction syndrome, ulnar shortening osteotomy, wafer procedure

## Abstract

**Background:**

Wrist pain on the ulnar side is often caused by ulnar impaction syndrome (UIS). Idiopathic UIS requires surgical treatment when conservative treatment fails. The 2 main surgical procedures used are the wafer procedure and ulnar shortening osteotomy (USO) of the metaphysis or diaphysis. This review aimed to analyze comparative studies of the 2 procedures in UIS to determine clinical outcomes and complications.

**Methods:**

One prospective and 5 retrospective comparison trials were retrieved from the PubMed, Embase, and Cochrane Library databases. The primary outcomes were treatment effectiveness; pain visual analog scale (VAS), disabilities of the arm, shoulder, and hand (DASH) score, Mayo wrist, and Darrow scores. The incidence of postoperative complications formed the secondary outcome.

**Results:**

The selected studies included 107 patients who underwent the wafer procedure (G1) and 117 patients who underwent USO (G2). The wafer procedure had the benefits of less postoperative immobilization and an early return to work. However, there were no significant differences in the postoperative pain improvement and functional scores. All 6 studies reported high total complication rates and reoperation with USO. The most frequent complication was implant-related discomfort or irritation; subsequent plate removal was the most common reason for a secondary operation.

**Conclusions:**

There was no difference in pain improvement or the postoperative functional score between the groups. Nevertheless, postoperative complications were the major pitfalls of USO. As the specialized shortening system advances further, a high-level study will be necessary to determine the surgical option in UIS.

## 1. Introduction

Pain on the ulnar side of the wrist is frequently caused by ulnar impaction syndrome (UIS). Excessive and repetitive weight bearing across the wrist ulnar aspect can lead to degeneration of the triangular fibrocartilage complex (TFCC), chondromalacia of the lunate and ulnar head, lunotriquetral ligament disruption, and eventually, severe arthritic changes of the ulnocarpal joint.^[1,2]^ Depending on the severity of the damage to these structures, UIS is divided into stages. The malunion or premature physeal arrest after distal radius fractures or other injuries can cause UIS. However, the condition is termed’ idiopathic’ UIS in patients with inherent ulnar positive variance or dynamic positive ulnar variance with wrist pronation and forceful grip without any trauma.^[3]^

Surgical treatment for idiopathic UIS is needed when conservative treatment fails (immobilization or radiocarpal corticosteroid injection). In patients with symptoms of ulnar impaction who are ulnar positive, decreasing mechanical collision due to ulnar recession is necessary to relieve symptoms. Therefore, the main surgical procedures used are joint resection, the wafer procedure, or osteotomy of the metaphysis or diaphysis.

The ulnar shortening osteotomy (USO) described in 1941 by Milch was the most commonly performed surgical procedure to treat UIS, and the extra-articular diaphyseal is typically fixed with a plate.^[4]^ This procedure preserves the joint capsules and ligaments around the wrist joint, and previous studies have reported good or excellent outcomes in 73% to 94% of patients.^[3,5,6]^ However, it is associated with complications. Simple complications include, for example, the bulk of the fixation plate, which often needs to be removed. Delayed union or nonunion—which have an incidence of approximately 10% in published studies^[7,8]^—are examples of more serious complications.

Described in 1992 by Feldon, the wafer procedure is partial excision of the distal ulna for decompression of the junction of the ulna, TFCC, lunate, and triquetrum, which retains the ligamentous attachments of the TFCC to the base of the styloid process and preserves the function of the distal radioulnar joint (DRUJ).^[9,10]^ Initially described as an open procedure, arthroscopy can be performed, especially when there is a central tear in the TFCC. The arthroscopic wafer procedure is suggested to be suitable for idiopathic UIS patients with a perforated TFCC,^[11,12]^ <4 mm of positive ulnar variance, and no instability of the DRUJ. It results in rapid recovery of daily life while avoiding USO-related complications.^[5,13,14]^

However, the limitation of a single surgeon infrequent experience has made comparative studies of available surgical treatments rare. No technique guarantees successful outcomes, presenting a therapeutic challenge. The choice of surgical treatment depends on the operating surgeon experience and personal preferences.

This review aimed to analyze comparative studies of the wafer procedure and USO in UIS to identify clinical outcomes and complications. The secondary goal was to perform a meta-analysis of the findings to compare the procedures.

## 2. Material and methods

### 2.1. Search strategy to identify studies

We followed the updated guidelines of the Preferred Reporting Items for Systematic Review and Meta-Analysis Protocols^[15]^ for this meta-analysis. The relevant institutional review board approved the study (approval no.: NHIMC 2021-09-005). Before we started the systematic review and meta-analysis, we registered our study design in PROSPERO, an international database of prospectively registered systematic reviews (identifier: CRD42021275536).

We searched the literature of the MEDLINE (PubMed), EMBASE, and Cochrane Library databases in July 2023. Supplementary Appendix A, http://links.lww.com/MD/J827 presents an overview of our search strategy. Articles reporting randomized, prospective, and case-control studies were included in the meta-analysis if they met the selection criteria. Only studies published in English were included due to difficulties in accurately translating non-English language studies. Selected articles were cross-checked for duplications.

### 2.2. Inclusion and exclusion criteria

Studies were included if they: were comparative studies (either prospective or retrospective) that evaluated postoperative outcomes after the wafer procedure or USO in UIS and had 1 or more clinical outcomes of pain score, function score, or postoperative complications. We considered that key points had not been performed if they were not clearly mentioned in a study.

The exclusion criteria were: study populations that underwent different surgical procedures to effect treatment; studies not reporting outcomes of interest; other forms of literature such as reviews, expert opinions, and basic science studies; and written in a language other than English.

### 2.3. Types of interventions

Patients were categorized into 2 groups based on the treatment modality for UIS: the wafer procedure (group G1) and USO (group G2).

### 2.4. Outcome measures and data extraction

The primary outcomes were pain and function scores. The visual analog scale (VAS) score was used to evaluate pain. This scale produces a score that ranges from 0 (no pain) to 10 (maximum pain). Three specific methods of assessing wrist function were used: the disabilities of the arm, shoulder, and hand (DASH) score,^[16]^ Mayo wrist score,^[17]^ and Darrow score.^[18]^ The Mayo wrist score and Darrow score are functional scoring systems in which scores are categorized as excellent, good, fair, or poor. To compare the Mayo wrist score and Darrow score, excellent and good were grouped into 1 group, and fair and poor into another. Odds were then calculated, and the meta-analysis was performed.

The secondary outcomes were postoperative complications, indicated by the incidence of reoperation and total complications, including implant discomfort or irritation and postoperative persistent clinical symptoms, including pain, ulnocarpal scar, infection, tendinopathy, carpal instability, ganglion, nonunion, re-fracture, nerve damage, and DRUJ instability. The reasons for the reoperations were addressed separately.

We extracted data from the included studies regarding the first author last name, year of publication, country where the study was conducted, study period, number of patients, patient age, sex ratio, side of the involved hand, Palmer classification, and follow-up duration. This data was collated into a single spreadsheet.

### 2.5. Study selection

After identifying possible literature to be included in this review, 2 authors (H.K.K. and J.H.L.) independently screened the titles and abstracts of each article against our inclusion and exclusion criteria. In cases where the abstract was unclear, the entire article was reviewed to determine whether the study met the inclusion criteria by the main authors (J.K.L. and Y.W.K.). After the initial screening, 2 independent authors (J.K.L. and J.H.L.) checked the full text of each potential article for eligibility.

Separately, each author recorded their screening results and why they had excluded a particular study. Where the authors disagreed, the 2 main authors (J.K.L. and Y.W.K.) made the final decision on which studies to include. If a study published data set was insufficient for our analysis, we emailed the study authors to obtain a complete set of the original data.

### 2.6. Quality assessment and publication bias

Two authors (J.W.H. and Y.W.K.) independently evaluated the quality and risk of bias in all the included studies. For the prospective comparative study, the grading of recommendations assessment, development, and evaluation (GRADE) system with 4 categories (high, moderate, low, and very low) was employed for quality levels and definition of a body of evidence. The risk of bias was measured with 5 domains according to Version 2 of the Cochrane tool for assessing risk of bias in randomized trials (RoB 2).^[19]^

For the retrospective comparative study, we used the Newcastle–Ottawa scale to independently evaluate all the studies included for this review for quality and risk of bias. This scale has 3 parameters—selection, comparability, and outcome—that each have subcategorized items. Selection has a maximum of 4 stars, comparability a maximum of 2 stars, and outcome (or exposure) a maximum of 3 stars. The outcomes are presented in Supplementary Appendix B, http://links.lww.com/MD/J830. We used Begg funnel plot^[20]^ and Egger test^[21]^ to assess publication bias. This process is explained in Appendix C.

### 2.7. Statistical analyses

We calculated the effect size as the weighted mean difference to analyze each continuous outcome. This value denotes the magnitude of differences between the groups being compared.^[22]^ For binary outcomes, effect sizes were calculated as the relative ratio (RR); studies were weighted according to the number of patients. All types of effect sizes are presented with their 95% confidence intervals (CIs).

Depending on the heterogeneity of the data, we used either a fixed- or random-effects model to quantify the pooled effect size of the studies included in this review. The chi-square (χ^2^) and I^2^ tests were used to evaluate heterogeneity between comparable studies; if *P > *.05 and I^2^ < 75, the fixed-effect model was used. All analyses were performed using STATA software (version 14.0; Stata Corporation, College Station, TX). Statistical significance was set at *P* < .05.

## 3. Results

### 3.1. Description of the included studies

**Figure 1.**
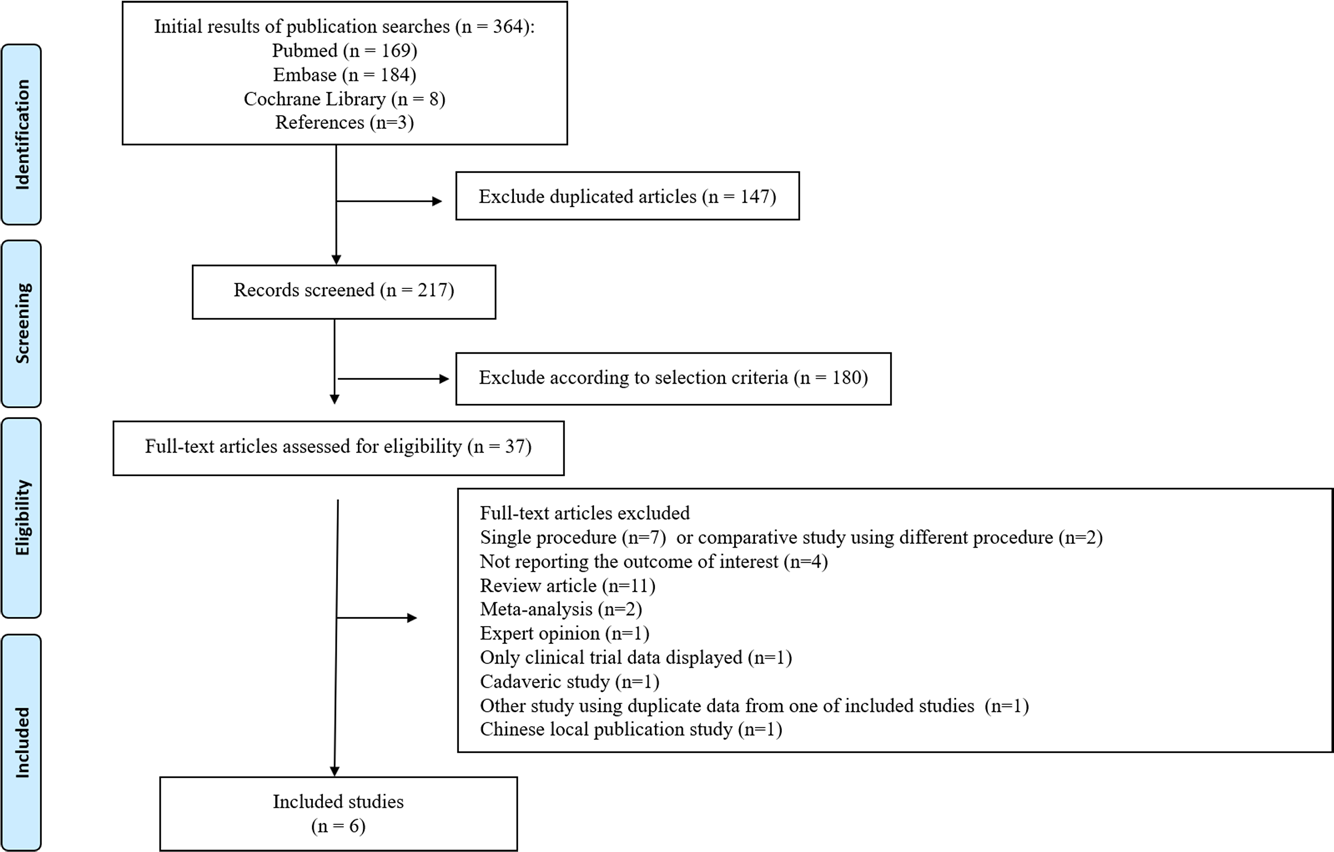
P preferred reporting items for systematic review and meta-analysis protocols flow diagram for clinical study selection.

A primary database search yielded 364 records. We excluded duplicates and screened 327 articles by title and abstract. As a result, 37 full-text articles were selected and reviewed for eligibility. We found 1 prospective randomized controlled study^[23]^ and 5 retrospective studies,^[5,6,24–26]^ which were included in the present study (Fig. 1). The selected studies included 107 patients who underwent the wafer procedure (G1) and 117 patients who underwent USO (G2). The characteristics of the included studies are summarized in Table 1. Detailed operative management of the included studies is summarized in Table 2.

**Table 1:** Characteristics of the included studies.

Study author (yr)	Country	Study design	Study Period	Sample number	Sex (male/female)	Age, SD or range	Palmer classification			Follow-up (mo)	Main outcome	Notes
							IA	IIC	IID			
Afifi, 2022	Egypt	Prospective	2014~2020	30 (G1) 30 (G2)	18/12 (G1) 26/4 (G2)	29 ± 4 (G1) 29 ± 6 (G2)		30 (G1) 30 (G2)		22 ± 5 (G1) 21 ± 5 (G2)	①②③⑤	Only prospective randomized control study
Auzias, 2021	France	Retrospective	1997 ~2017	24 (G1) 9 (G2)	NA	44 ± 12 (G1) 39 ± 4 (G2)	NA			55 ± 4 (G1) 103 ± 8 (G2)	①②⑤	9 patients of 2ndary UIS
Oh, 2018	South Korea	Retrospective	2009.5~2014.6	19 (G1) 23 (G2)	8/11 (G1) 9/14 (G2)	53 ± 7 (G1) 53 ± 6 (G2)		8 (G1) 7 (G2)	11 (G1) 16 (G2)	34 ± 12 (G1) 36 ± 11.6 (G2)	①②③⑤	- Raw data provided, - Serial follow up study of 3/6/16/and 24 mo
Smet, 2014	Belgium	Retrospective	NA	12 (G1) 28 (G2)	4/8 (G1) 6/22 (G2)	46 (31~66) (G1) 38 (16~61) (G2)	NA			NA (G1) 29 (7–60) (G2)	①②③⑤	17 of 28 ulnar shortening patients were 2ndary procedure after arthroscopic TFCC debridement
Bernstein, 2004	United States	Retrospective	1988~2000	11 (G1) 16 (G2)	5/6 (G1) 8/8 (G2)	37 (24~61) (G1) 38 (19~65) (G2)	6 (G1) 10 (G2)	2 (G1) 2 (G2)	3 (G1) 4 (G2)	21 (7~61) (G1) 15 (7~58) (G2)	③④⑤	9 of 11 AWP and 12 of 16 USO cases involved Workers’ Compensation
Constantine 2000	United States	Retrospective	1990~1996	11 (G1) 11 (G2)	3/8 (G1) 5/6 (G2)	46 (G1) 35 (G2)	NA			26 (G1) 46 (G2)	③④⑤	Open Wafer procedure

① visual analog scale score, ② disabilities of the arm, shoulder and hand score, ③ Mayo wrist score, ④ Darrow score, and ⑤ post-operative complication.

G = group, G1 = arthroscopic wafer procedure, G2 = ulnar shortening osteotomy, NA = not addressed, SD = standard deviation, TFCC = triangular fibrous cartilage complex, UIS = ulnar impaction syndrome.

**Table 2:** Details of 2 procedures.

Author, year	Group	Pre-Op ulnar variance (mm, SD or range)	Operating surgeon	Arthroscopy combined	Procedure detail	Post-op ulnar variance (mm, SD or range)	Immobilization method (duration)	Union time after USO (average weeks, range)	Return to work, SD (±) or range (~)
					Wafer: instrument/resection target				
					USO: OT location/direction/shortening target/plate position/screw size/plate details				
Afifi, 2022	Wafer	2.2 (0.6)	Single surgeon	Yes	2.9 mm burr/2~4 mm resection	−0.3 (0 ~ −1)	Below-elbow orthosis (2 wk) => thermoplastic orthosis (2 wk)		57.7 ± 6.5 d
	USO	2.4 (0.4)			Distal Diaphysis (2/3)/Transverse/2~4 mm/Ulnar/3.5 mm/6 hole DCP	−0.4 (0 ~ −1)		12	117 ± 8.6 d
Auzias, 2021	Wafer	0.9 (0.8)	Single surgeon	Yes	3.5 mm burr/2~3 mm resection	NA	Short arm volar splint (10 d)		3.75 mo
	USO	2.0 (2.0)		None	Diaphysis/Oblique/NA/Volar/3.5 mm/LC-DCP (Before 2010) or Synthes cutting guided 7 or 8 hole DCP	NA	Long arm volar splint/(4 wk)	NA	7.86 mo
Oh, 2018	Wafer	3.0 (0.6)	Single surgeon	Yes	2.9 mm or 3.5 mm burr/2~4 mm resection with DRUJ sparing	0.10 (0.6)	Short arm volar splint (2 wk)		NA
	USO	2.9 (0.6)			Distal third diaphysis/Transverse/2~4 mm/Volar/3.5 mm/locking compression plate or specialized ulnar shortening system	−0.1 (0.8)	Short arm volar splint (2 wk)	6.8	
Smet, 2014	Wafer	2.7 (−3.5~5)	NA	None	Arthroscopic bur/2 mm	Unchanged	Only for pain control		6.1 (0~26) mo
	USO	1.7 (−1 ~4)		Yes	Diaphysis/Transverse (19), Oblique (9)/3.5 mm/NA/3.5 mm/AO DCP	−1.8 (−4 ~ 0.5)	None	NA	7 (0~30) mo
Bernstein, 2004	Wafer	1.54 (0.5~3)	Single surgeon	Yes	holmium:yttrium-aluminum- garnet (Ho:YAG) Laser then, 3.5-mm mini-burr or 2.9 mm barrel abrader/1~2 mm	−1 (−0.5 ~ −2)	Volar wrist splint (6 wk)		7.3 (1.5~16) mo
	USO	1.45 (0.5~3)	Other surgeons		Diaphysis/Oblique/3 mm/NA/3.5 mm/Rayhack device (6), 7-hole DCP or LC-DCP plate (Syntehs, paoli, PA) (10)	−1 (<−0.5~−2.0)	Short or long arm splint (2 wk) => Short arm thermoplastic splint (4 wk)	12 (10~21)	7.5 (2.0~16) mo
Constantine.2000	Wafer	1.5 (0~4)	Single surgeon	None	Open resection/NA	1	sugar tong splint (2 wk) => long arm cast (2 wk)		NA*
	USO	2.5 (1~5)	Two different surgeons		Diaphysis/Transverse/NA/NA/3.5 mm/6-hole LC-DCP plate (Syntehs, paoli, PA	1	Long arm splint (2 wk) => long arm cast (2 wk)	12	NA*

DCP = dynamic compression plate, DRUJ = distal radioulnar joint, LC-LCP = limited contract-locking compression plate, NA = not addressed, OT = osteotomy, SD = standard deviation, USO = ulnar shortening osteotomy.

* Constantine 2000 reported rates of return to work: Wafer: 8 (72.7%), USO: 9 (81.8%).

### 3.2. Quality assessment and publication bias

There was only 1 prospective randomized controlled study included.^[23]^ Although the study adhered to the principles of random allocation and blinding the operating surgeon and measurement observer, there was a limitation in the small sample size. According to the Version 2 of the Cochrane tool for assessing risk of bias in randomized trials (RoB 2), it was graded as low.

In terms of methodological quality in retrospective comparative studies, the mean value of the awarded star was 7.2 (two studies had 9 stars,^[5,25]^ 1 study had 8 stars,^[6]^ 1 study had 7 stars,^[26]^ and 1 study had 3 stars,^[24]^ Supplementary Appendix B, http://links.lww.com/MD/J830). The Begg funnel plot appeared asymmetrical, but the *P* value for bias was not significant (Supplementary Appendix C, http://links.lww.com/MD/J832).

### 3.3. Primary outcomes

**Figure 2.**
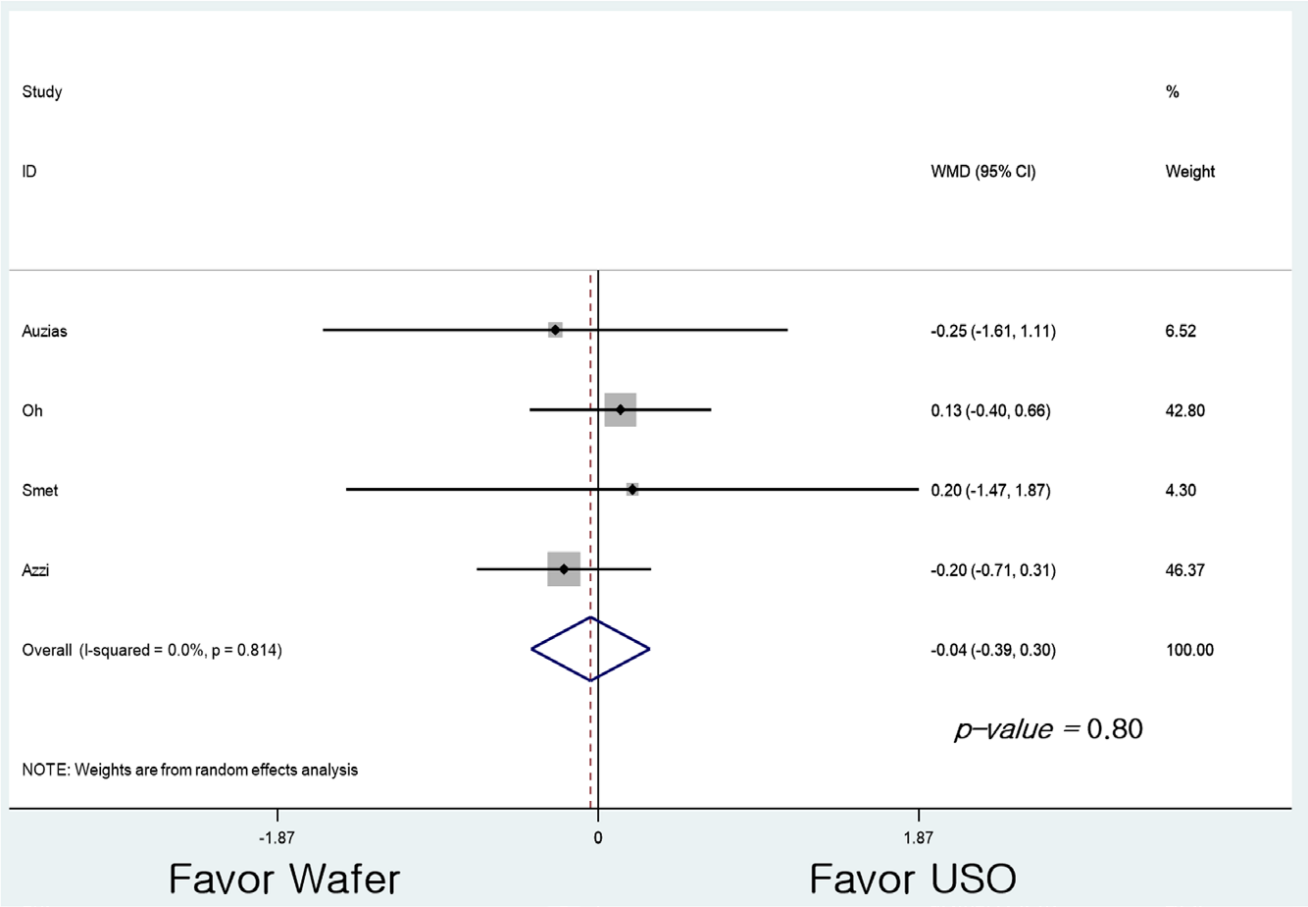
Forest plots of the studies showing visual analog scale outcomes.

Four of the studies included in this review evaluated pain.^[23–26]^ At the final follow-up, there was no difference in pain VAS comparison between G1 (n = 85) and G2 (n = 90) (95% CI: −0.391 to 0.302, *P* = .80; Fig. 2).

**Figure 3.**
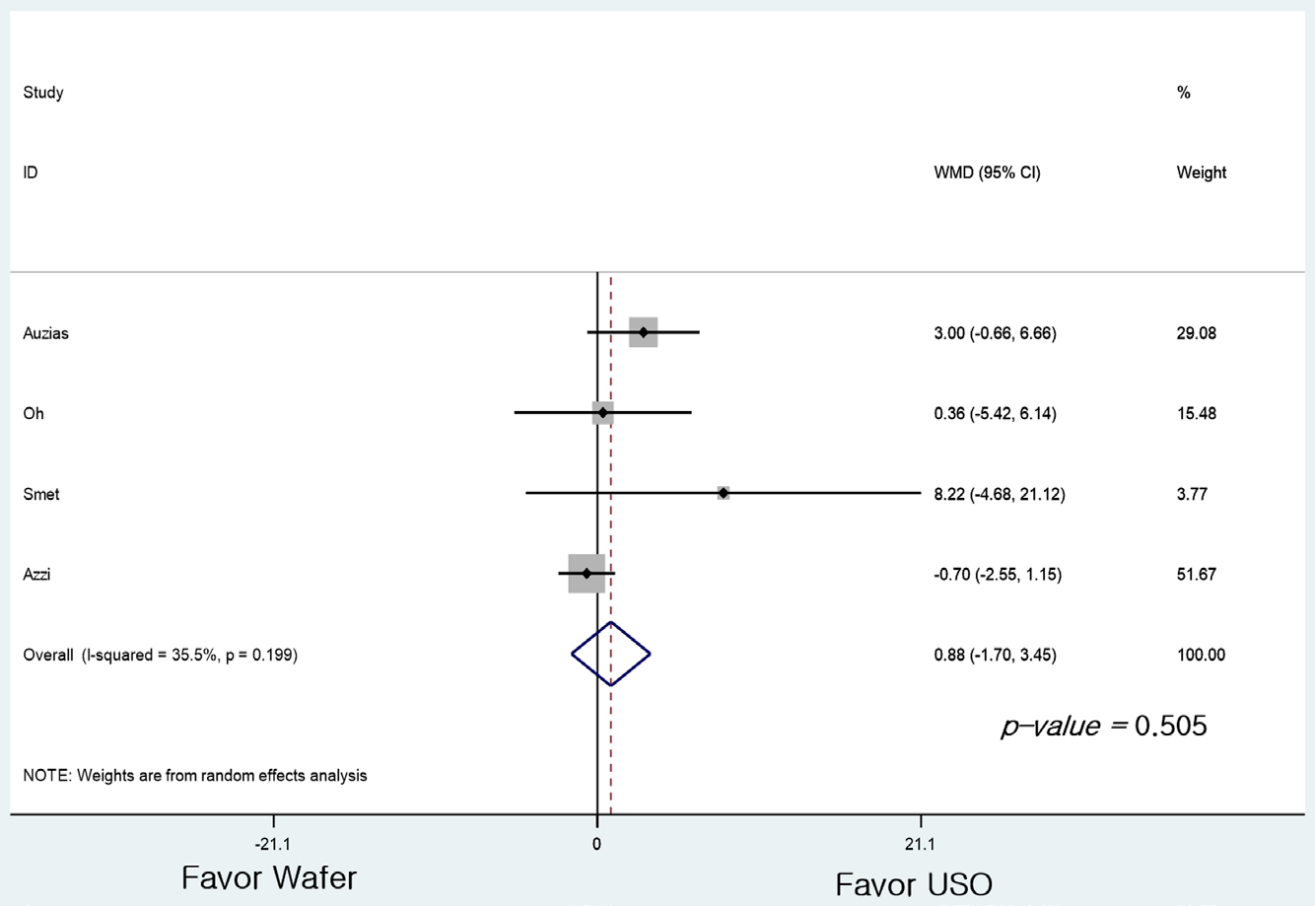
Forest plots of the studies showing the disabilities of the arm, shoulder, and hand score.

The DASH score was compared between 4 studies, and the findings were similar to the VAS assessment.^[23–26]^ G2 had a DASH score 0.88 points lower than G1 without significant difference (95% CI: −1.700 to 3.453 points, *P* = .505; Fig. 3).

**Figure 4.**
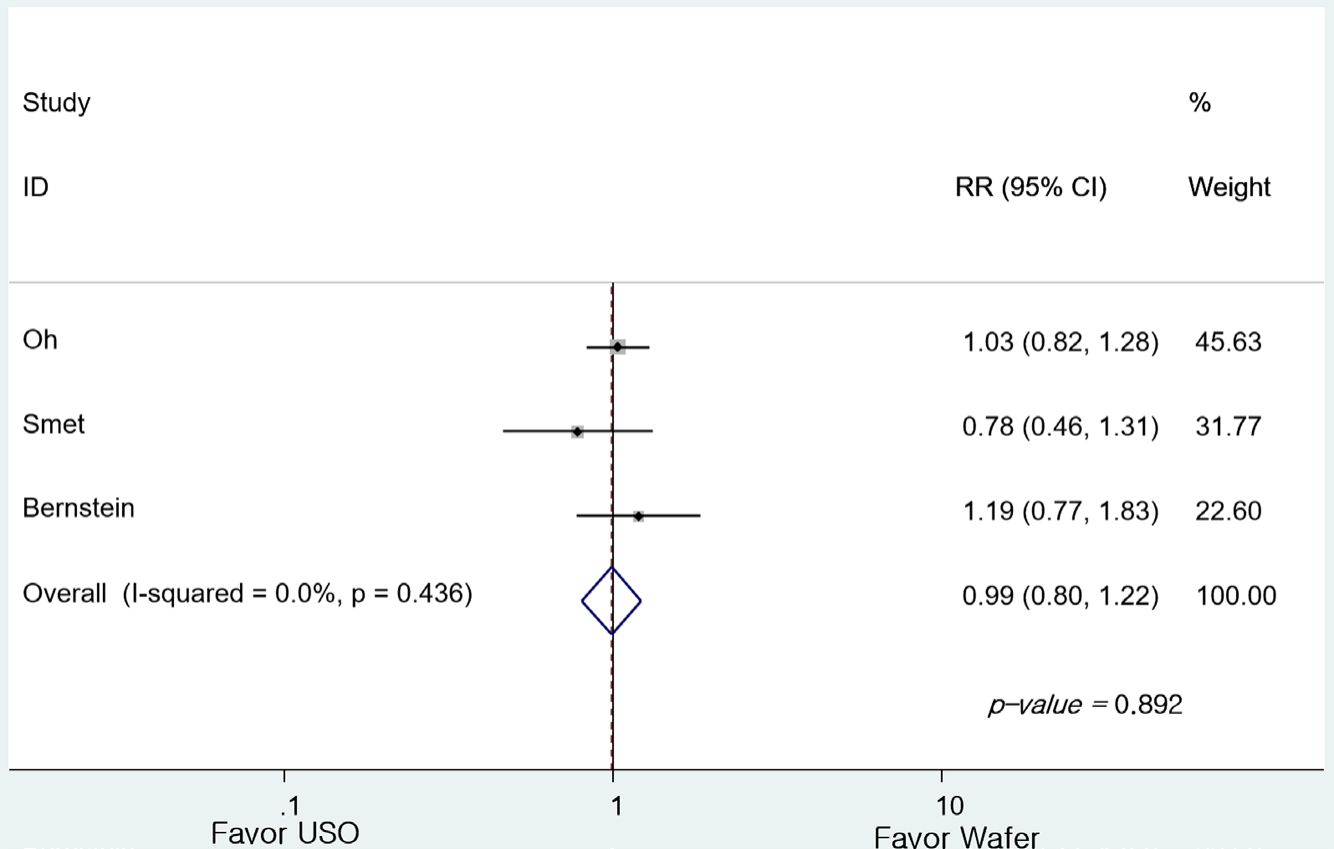
Forest plot of the differences in the Mayo wrist score between the groups (reported as grade).

**Figure 5.**
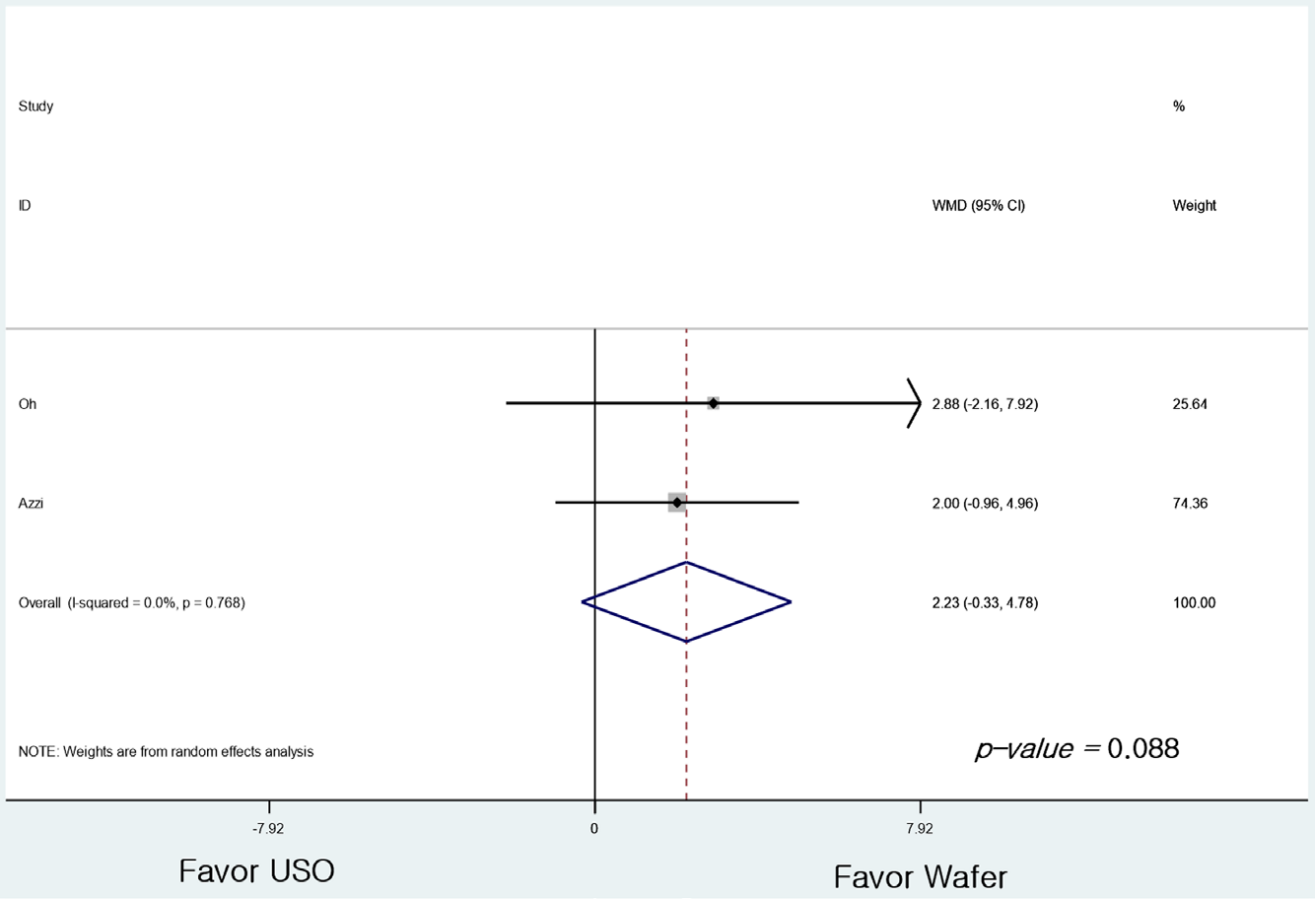
Forest plot of the differences in the Mayo wrist score between the groups (reported as average score).

Regarding the Mayo wrist score, the 3 studies^[5,24,25]^ reported outcomes as grades, and 2 studies^[23,25]^ as mean values. In a meta-analysis comparing 4 grades reclassified into 2 categories, there was no difference between G1 (n = 42) and G2 (n = 67) (RR = 0.986, 95% CI: 0.799–1.216, *P* = .892; Fig. 4). There was no difference between the 2 groups even when the results of the 2 studies presented as average values were compared (95% CI: −0.327 to 4.779 points, *P* = .088; Fig. 5).

**Figure 6.**
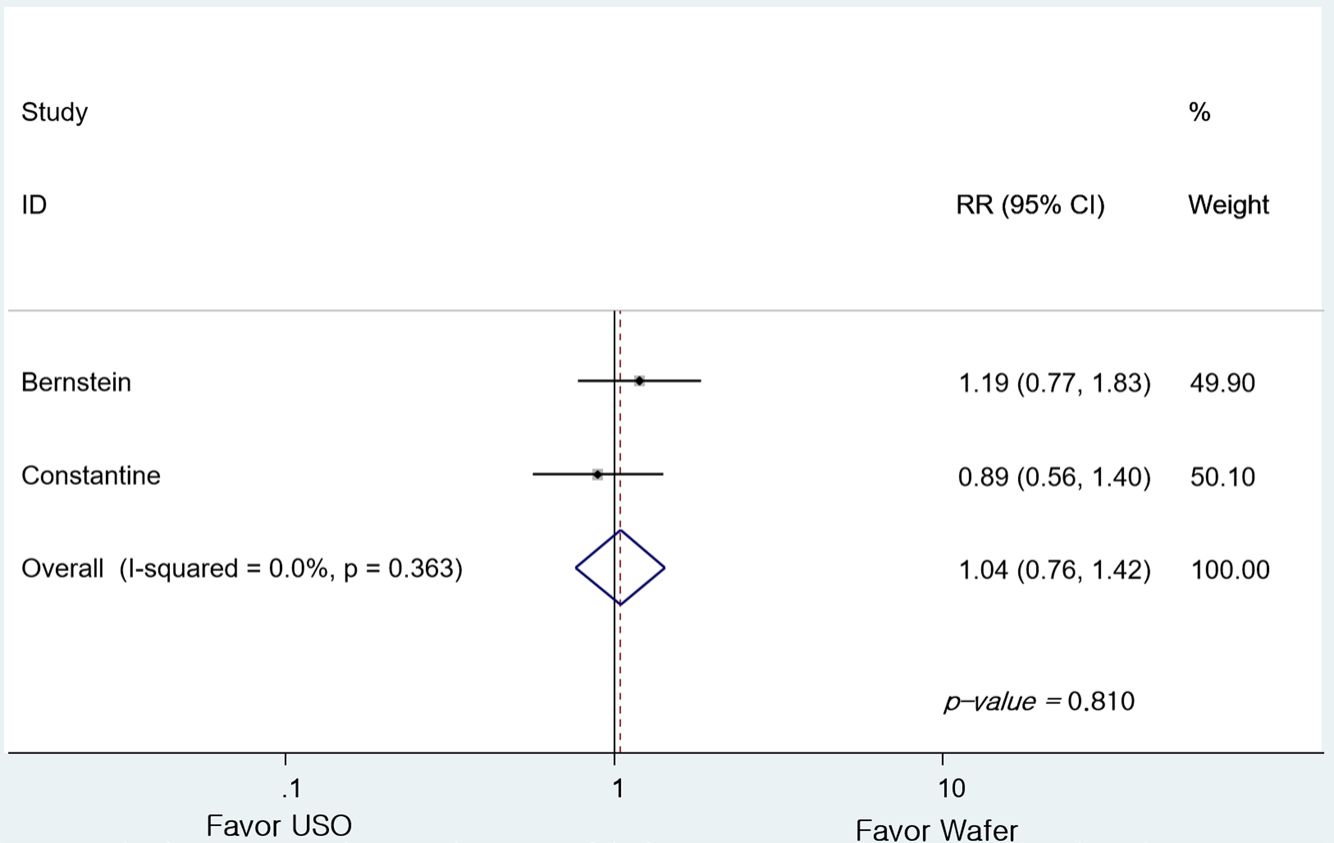
Forest plot of the differences in Darrow score between the groups.

Darrow score was compared between 2 studies.^[5,6]^ In a meta-analysis comparing 4 grades reclassified into 2 categories, there was no difference between G1 (n = 22) and G2 (n = 27) (RR = 1.039, 95% CI: 0.760–1.421, *P* = .810; Fig. 6).

### 3.4. Secondary outcomes

The overall complication and reoperation rates are summarized in Tables 3 and 4, respectively. There were studies that reported multiple complications in 1 operation, and in this case, the total number of complications was higher than the number of operations; therefore, meta-analysis could not be performed.^[5,24]^

**Table 3:** Total complications.

Author, year	Group	No procedures	Total number	Details											
				Hardware discomfort or irritation	Cold intolerance	Persistent discomfort or pain	Ulnocarpal scar	Infection	Tendinopathy	Carpal instability	Ganglion	Nonunion	Re-fracture	Ulnar nerve dorsal sensory branch paresthesia	DRUJ problems
Afifi, 2022	Wafer	30	1											1	
	USO	30	12	12											
Auzias, 2021	Wafer	24	3			2			1						
	USO	9	7	5								1	1		
Oh, 2018	Wafer	19	2			1		0	1				0		
	USO	23	8	6				0	0				2		
Smet, 2014	Wafer	12	2			1				1					
	USO	28	29*	22		1						3			3
Bernstein, 2004	Wafer	11	5				1	1	2		1				
	USO	16	18*	9		1	1		7						
Constantine. 2000	Wafer	11	1			1									
	USO	11	7	5	2										

DRUJ = distal radioulnar joint, op = operation, USO = ulnar shortening oteotomy.

* Multiple complications occurred in 1 unit, resulting in a greater number of complications than the total number.

**Table 4:** Reoperation complications.

Author, year	Group	No procedures	Reoperation	
			Number	Reasons (n, %)
Afifi, 2022	Wafer	30	0	
	USO	30	11	Removal from hardware irritation (11, 36.7%)
Auzias, 2021	Wafer	24	3	ECU stabilization for tendon instability (1, 4.1%) styloid excision for Ulnar styloid nonunion (1, 4.1%) Wrist denervation (1, 4.1%)
	USO	9	7	Plate removal (5, 55.6%) Nonunion (1, 11.1%) Refracture after plate removal (1, 11.1%)
Oh, 2018	Wafer	19	1	USO conversion (1, 5.2%))
	USO	23	15	Plate removal (13, 56.5%) Refracture after plate removal (2. 8.6%)
Smet, 2014	Wafer	12	2	USO conversion (1, 8.3%) Blatt capsulodesis for scapholunate instabilty (1, 8.3%)
	USO	28	30*	Plate removal (22, 78.6%) Nonunion (3, 10.7%) Sauve-Kapanji op (1, 3.6%) Cubital tunnel release (1, 3.6%) DRUJ stabilization (1, 3.6%) DRUJ arthrolysis (1, 3.6%) Wrist arthrodesis (1, 3.6%)
Bernstein, 2004	Wafer	11	1	Arthroscopic debridement (1, 9%)
	USO	16	10	Plate removal (9, 56.3%) Arthroscopic debridement (1, 6.25%)
Constantine. 2000	Wafer	11	1	Distal ulnar resection (1, 9%)
	USO	11	5	Plate removal (5, 45.5%)

DRUJ = distal radioulnar joint, op = operation, USO = ulnar shortening oteotomy.

* Multiple complications occurred in 1 unit, resulting in a greater number of complications than the total number.

All 5 included studies reported high total complication rates and reoperation in G2.^[5,6,23–26]^ The most common complication was implant-related discomfort or irritation, and subsequent plate removal was the reason for the largest number of secondary operations performed, amounting to 50%^[5,6,25,26]^ and 78% of cases, respectively.^[24]^

## 4. Discussion

This systematic review of idiopathic UIS found comparable functional scores and complications between the wafer procedure and USO. Our results can be summarized as follows. There was no significant difference in the postoperative pain improvement and functional scores between these 2 types of procedures. The wafer procedure had the benefits of less postoperative immobilization and an early return to work. In addition, the frequency of complication occurrence was higher with USO than with the wafer procedure.

Until a prospective randomized controlled trial study was reported in 2022, all were retrospective comparison studies for this subject.^[23]^ The significance of this study is that a meta-analysis was performed by adding one recently published prospective comparison study to the previous 5 retrospective comparison studies. Nevertheless, most studies, including prospective studies, reported similar results with no difference in the degree of clinical outcome improvement between the 2 groups.

We could not perform a meta-analysis of range of motion (ROM) and grip power. In a prospective study by Afifi et al, it was reported that there were no differences between the 2 groups in all joint movements.^[23]^ Auzias et al,^[26]^ Oh et al,^[25]^ and Constantine et al^[6]^ also described that there was no difference in ROM at the final follow-up though their study did not suggest an accurate ROM angle. In Oh et al’s study, which was the only one that was serially measured at 3, 6, 12, and 24 months, there was no difference in flexion-extension arcs, radial and ulnar deviation arcs, or supination arcs according to the postoperative period.^[25]^ No study has reported an increase in the joint range after surgery, but no study has reported a serious decrease in the joint ROM.

In the same way, the grip power analysis could not be performed because the existing studies did not present feasible results for comparison. Afifi et el., Oh et al and Bernstein et al reported no difference between the 2 groups after each operation.^[5,23,25]^ However, Auzias et al^[26]^ and Constantine et al^[6]^ reported that USO showed higher grip power than the wafer procedure, although the differences were not statistically significant.

Since the first introduction of the wafer procedure in 1992,^[9]^ there has been controversy regarding the superiority of the wafer procedure over USO.^[5,13,27]^ The advantages of the wafer procedure include less surgical pain, less disruption of the dorsal radiocarpal and radioulnar capsules, and no requirement for implant removal or bony union.^[27]^ Because the wafer procedure is less invasive and does not require fracture fusion as USO does, it has the advantages of a shorter immobilization period after the operation and a quick return to work. Three studies reported the average time required for the bone union from 6 to 12 weeks after USO.^[5,6,23,25]^ Despite no clinical differences, Afifi et al,^[23]^ Oh et al,^[25]^ and Smet et al^[24]^ also reported a significantly lower duration of work time off in the wafer procedure group than in the USO group. Auzias et al noted that the only difference was that the patients in the arthroscopic wafer group returned to work faster than those in the USO group.^[26]^ Although there are no absolute indications or contraindications for each procedure, there have been arguments claiming limited indications for the arthroscopic wafer procedure, including < 4 mm of positive ulnar variance, Palmer type 2C or 2D lesions of the TFCC, stable DRUJ and/or lunotriquetral joint, and no evidence of instability or osteoarthritis at the DRUJ or ulnocarpal joint.^[5,13,14,25,28]^ Careful selection of patients is essential to ensure satisfactory outcomes of the wafer procedure. In the studies by Oh et al and Smet et al, 1 person in each wafer procedure group was converted to USO as a secondary operation with persistent symptoms even after undergoing the wafer procedure.^[24,25]^

In addition, this systematic review showed more complications with USO than with the wafer procedure, although we could not perform a meta-analysis due to statistical difficulty. In contrast to the wafer procedure, USO requires adequate osteotomy, bone shortening, instrument fixation, and subsequent bone union. Various complications can occur during each surgical procedure, including symptomatic implant irritation (rates of 0%–45%), delayed union or nonunion, secondary DRUJ arthritis, and re-fracture after removal of the fixation device.^[6–8,29]^ Despite the good clinical outcomes of USO, it has critical shortcomings. Most notably, delayed union or nonunion has been noticed with a variable incidence (0–12.7%) and remains unresolved.^[7,8,29]^ Many factors, including patient factors, surgeon skills, and technique decisions, may contribute to this. Among the included studies, 2 reported the occurrence of nonunion and that secondary operation was required (Auzias et al, 1/9 [11.1%] and Smet et al, 3/28 [10.7%], respectively).^[24,26]^ Auzias et al,^[26]^ Smet et al,^[24]^ and Constantine et al^[6]^ reported in their studies that osteotomy was performed free hand and stabilized with a low contact-dynamic compression plate. Bernstein et al^[5]^ and Oh et al^[25]^ who did not report nonunion, noted that a specialized ulnar shortening system was used in some patients, and surgical procedures for guide cutting were described. Compared to the popular freehand osteotomy technique, several companies started to launch specialized shortening osteotomy systems since 2005.^[30–33]^ These systems facilitate USO with precise and parallel osteotomy, and subsequently, the incidence of delayed or nonunion is expected to decrease.^[32–36]^ Furthermore, advancement and revision in the future will enable more stable and consistent USO. Nevertheless, it is possible that other technical factors, such as periosteal stripping, soft tissue dissection, and postoperative management, also contributed to the difference in the nonunion rate regardless of the orientation of the osteotomy.^[37]^ Therefore, there is a limit to simply comparing the frequency of nonunion depending on the application of the osteotomy method or the specialized ulnar shortening system, and further research is needed.

The most common complication of USO is plate discomfort or irritation, and significant plate removal is required for secondary surgery. A recent study reported that the specialized shortening system reduced the plate removal rates compared to the conventional freehand technique.^[32]^ In terms of plate position affecting plate removal, there have been controversial arguments when it is applied to the volar, ulnar, and dorsal sides.^[38–40]^ Nevertheless, to avoid plate-related problems, a low-profile plate is recommended.^[38,39]^ Current knowledge of plate-related clinical symptoms is an unsolved problem in USO.

There are several limitations to this systematic review and meta-analysis. First, only 1 prospective comparative study between the 2 procedures was performed, and the included studies were all conducted with small sample sizes. Second, the heterogeneity of the methods used in the UIS literature inhibited the statistical analysis. Lastly, various techniques based on the surgeon experience and instruments can affect clinical outcomes and complications after UIS. Hence, the advancement in specialized shortening instruments may have positive effects on USO in the future.

## 5. Conclusions

There was no difference in pain improvement or the postoperative functional score between the procedures in our meta-analysis. Nevertheless, postoperative complications were the major pitfalls of USO. As the specialized shortening system advances further, a high-level study in the future is necessary to determine the surgical option in UIS.

## Acknowledgments

We would like to thank Yun-Rak Choi and Won-Taek Oh, main authors of one included study, for providing raw data.

## Author contributions

**Conceptualization:** Joong Won Ha, Jun-Ku Lee.

**Data curation:** Young Woo Kwon, Sujung Lee, Jinho Lee, Chae Kwang Lim.

**Investigation:** Sujung Lee.

**Methodology:** Sujung Lee, Hyunsun Lim.

**Resources:** Sujung Lee.

**Software:** Hyunsun Lim.

**Supervision:** Jun-Ku Lee.

**Validation:** Hyunsun Lim, Jun-Ku Lee.

**Visualization:** Hyunsun Lim.

**Writing – original draft:** Joong Won Ha, Young Woo Kwon.

**Writing – review & editing:** Chae Kwang Lim, Jun-Ku Lee.

## Supplementary Material






