# Supplementary material for: Is ulnar shortening osteotomy or the wafer procedure better for ulnar impaction syndrome?: A systematic review and meta-analysis
Source: Medicine (Baltimore). 2023 Sep 29;102(39):e35141. doi: 10.1097/MD.0000000000035141 (PMC10545262; doi:10.1097/MD.0000000000035141)
Supplement: Supplementary file 1 [file medi-102-e35141-s001.docx]

**Queries**

1. Population
   ulnar impaction (ulnocarpal impaction or ulnar abutment) syndrome
2. Intervention and Control
   Ulnar shortening osteotomy vs Wafer procedure
3. Outcome

Pain, function, and complication

1. Study type

None

**Pubmed 20230704 – 169 articles**

("ulnar impaction syndrome"[All Fields] OR "uis"[All Fields] OR ("ulnocarpal"[All Fields] AND ("impingement"[All Fields] OR "impingements"[All Fields] OR "impingment"[All Fields]) AND ("syndrom"[All Fields] OR "syndromal"[All Fields] OR "syndromally"[All Fields] OR "syndrome"[MeSH Terms] OR "syndrome"[All Fields] OR "syndromes"[All Fields] OR "syndrome s"[All Fields] OR "syndromic"[All Fields] OR "syndroms"[All Fields])) OR "ulnocarpal impaction syndrome"[All Fields] OR "uci"[All Fields] OR "ulna impaction syndrome"[All Fields] OR ("ulnocarpal"[All Fields] AND ("abutment"[All Fields] OR "abutment s"[All Fields] OR "abutments"[All Fields]) AND ("syndrom"[All Fields] OR "syndromal"[All Fields] OR "syndromally"[All Fields] OR "syndrome"[MeSH Terms] OR "syndrome"[All Fields] OR "syndromes"[All Fields] OR "syndrome s"[All Fields] OR "syndromic"[All Fields] OR "syndroms"[All Fields]) AND "or"[All Fields] AND (("ulnar artery"[MeSH Terms] OR ("ulnar"[All Fields] AND "artery"[All Fields]) OR "ulnar artery"[All Fields] OR "ulnar"[All Fields]) AND ("abutment"[All Fields] OR "abutment s"[All Fields] OR "abutments"[All Fields]) AND ("syndrom"[All Fields] OR "syndromal"[All Fields] OR "syndromally"[All Fields] OR "syndrome"[MeSH Terms] OR "syndrome"[All Fields] OR "syndromes"[All Fields] OR "syndrome s"[All Fields] OR "syndromic"[All Fields] OR "syndroms"[All Fields]))) OR "uas"[All Fields] OR ("brachyura"[MeSH Terms] OR "brachyura"[All Fields] OR "ucas"[All Fields])) AND ((("ulnar artery"[MeSH Terms] OR ("ulnar"[All Fields] AND "artery"[All Fields]) OR "ulnar artery"[All Fields] OR "ulnar"[All Fields]) AND ("shorted"[All Fields] OR "shortening"[All Fields]) AND ("osteotomie"[All Fields] OR "osteotomied"[All Fields] OR "osteotomy"[MeSH Terms] OR "osteotomy"[All Fields] OR "osteotomies"[All Fields])) OR (("ulna"[MeSH Terms] OR "ulna"[All Fields]) AND ("shorted"[All Fields] OR "shortening"[All Fields]) AND ("osteotomie"[All Fields] OR "osteotomied"[All Fields] OR "osteotomy"[MeSH Terms] OR "osteotomy"[All Fields] OR "osteotomies"[All Fields])) OR "uso"[All Fields] OR "wafer procedure"[All Fields] OR "wafer procedure*"[All Fields] OR "wafer resection"[All Fields] OR "wafer resection*"[All Fields])

**EMbase 20230704 - 184 articles**

**
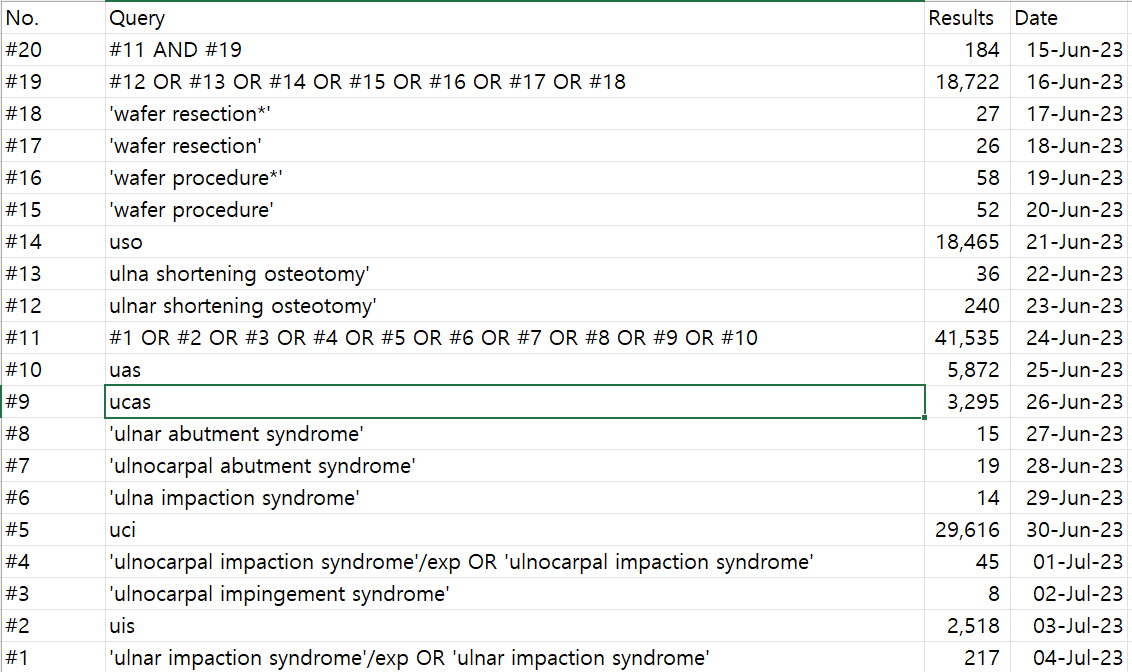
**

**Cochrane 20230704 – 2 articles**

| Search Name: |  |  |
| --- | --- | --- |
| Date Run: | 06/09/2021 23:26:37 |  |
| Comment: |  |  |
|  |  |  |
| ID | Search | Hits |
| #1 | ("ulnar impaction syndrome"):ti,ab,kw OR ("ulnocarpal impingement syndrome"):ti,ab,kw OR ("ulnocarpal impaction syndrome"):ti,ab,kw OR ("ulna impaction syndrome"):ti,ab,kw | 11 |
| #2 | ("ulnocarpal abutment syndrome"):ti,ab,kw OR ("ulnar abutment syndrome"):ti,ab,kw | 2 |
| **#3** | **#1 OR #2** | **12** |
| #4 | ("ulnar shortening osteotomy"):ti,ab,kw OR ("ulna shortening osteotomy"):ti,ab,kw OR ("wafer procedure"):ti,ab,kw OR ("Wafer procedure*"):ti,ab,kw | 8 |
| #5 | ("wafer resection"):ti,ab,kw OR ("wafer resection*"):ti,ab,kw | 0 |
| #6 | #4 OR #5 | 2 |
| **#7** | **#3 AND #6** | **8** |

**Hand searching – 3 articles**
