# Supplementary material for: Is ulnar shortening osteotomy or the wafer procedure better for ulnar impaction syndrome?: A systematic review and meta-analysis
Source: Medicine (Baltimore). 2023 Sep 29;102(39):e35141. doi: 10.1097/MD.0000000000035141 (PMC10545262; doi:10.1097/MD.0000000000035141)
Supplement: Supplementary file 2 [file medi-102-e35141-s002.docx]

| Study (year) | Selection | Comparability | Exposure | Total |
| --- | --- | --- | --- | --- |
| Auzias, 2021 | ☆☆☆☆ | ☆ | ☆☆ | 7 |
| Oh, 2018 | ☆☆☆☆ | ☆☆ | ☆☆☆ | 9 |
| Smet, 2014 | ☆☆ |  | ☆ | 3 |
| Bernstein, 2004 | ☆☆☆☆ | ☆☆ | ☆☆☆ | 9 |
| Constantine.2000 | ☆☆☆☆ | ☆ | ☆☆☆ | 8 |

**Appendix B**. Methodological quality assessment of included retrospective studies measured by Newcastle-Ottawa Scale
