# Supplementary figures and images for: Is ulnar shortening osteotomy or the wafer procedure better for ulnar impaction syndrome?: A systematic review and meta-analysis
Source: Medicine (Baltimore). 2023 Sep 29;102(39):e35141. doi: 10.1097/MD.0000000000035141 (PMC10545262; doi:10.1097/MD.0000000000035141)

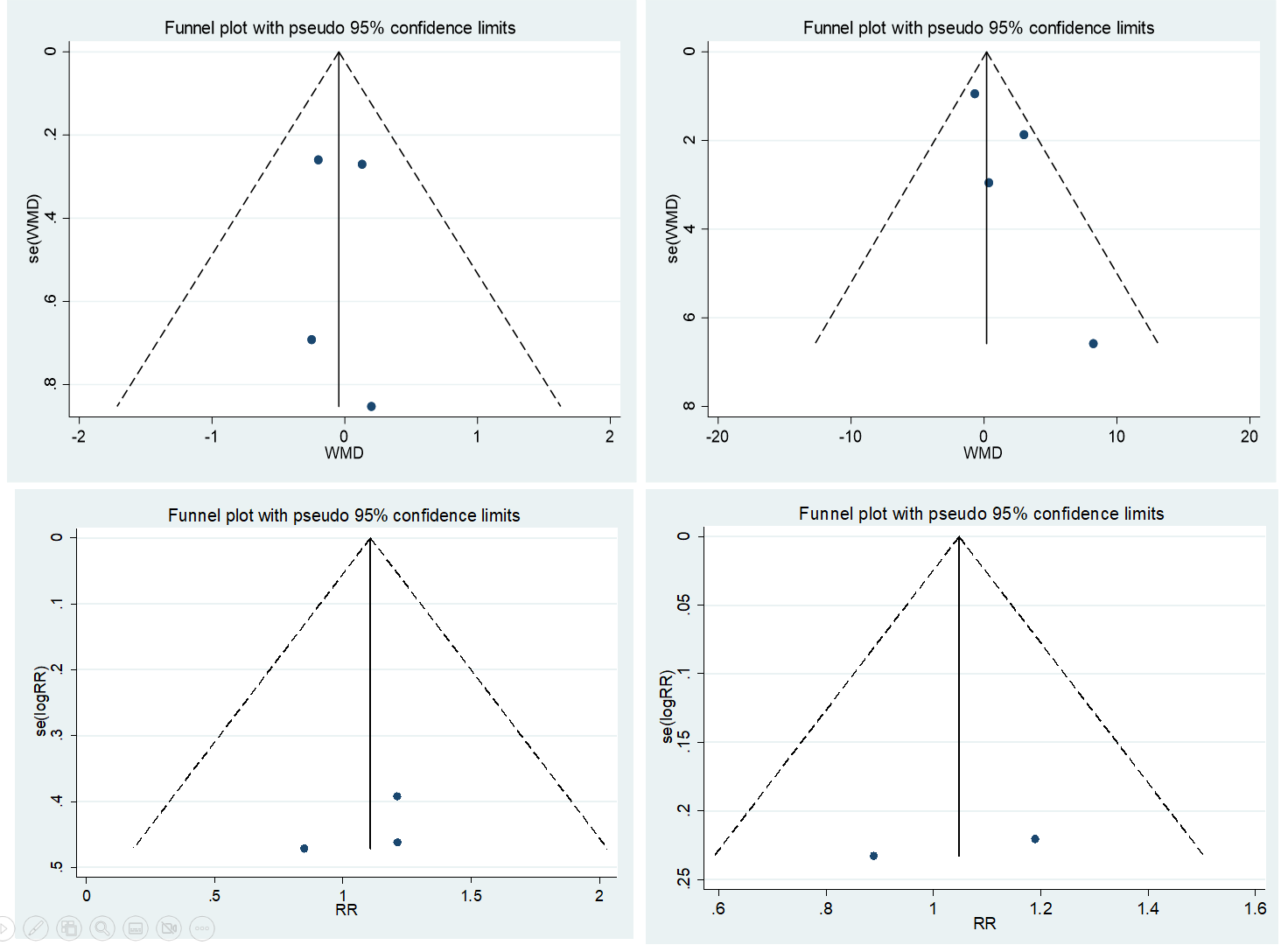

Supplement: Supplementary file 3 [file medi-102-e35141-s003.tif]
